# Metabolic activity via ^18^F-FDG PET/CT is predictive of microsatellite instability status in colorectal cancer

**DOI:** 10.1186/s12885-022-09871-z

**Published:** 2022-07-22

**Authors:** Jinling Song, Zhongwu Li, Lujing Yang, Maomao Wei, Zhi Yang, Xuejuan Wang

**Affiliations:** 1 Department of Nuclear Medicine, The Cancer Hospital of the University of Chinese Academy of Sciences (Zhejiang Cancer Hospital), Institute of Basic Medicine and Cancer (IBMC), Chinese Academy of Sciences, Hangzhou, Zhejiang 310022 China; 2 Key laboratory of Carcinogenesis and Translational Research (Ministry of Education), Department of Pathology, Peking University Cancer Hospital & Institute, Beijing, 100142 China; 3 Key Laboratory of Carcinogenesis and Translational Research (Ministry of Education), NMPA Key Laboratory for Research and Evaluation of Radiopharmaceuticals (National Medical Products Administration), Department of Nuclear Medicine, Peking University Cancer Hospital & Institute, Beijing, 100142 China

**Keywords:** Colorectal cancer, Microsatellite instability, Metabolic tumor volume, Positron emission tomography

## Abstract

**Purpose:**

Identification of microsatellite instability high (MSI-H) colorectal cancer (CRC) is crucial for screening patients most likely to benefit from immunotherapy. We aim to investigate whether the metabolic characteristics is related to MSI status and can be used to predict the MSI-H CRC.

**Methods:**

A retrospective analysis was conducted on 420 CRC patients who were identified via [^18^F]fluorodeoxyglucose (^18^F-FDG) positron emission tomography (PET)/computed tomography(CT) prior to therapy. Maximum standardized uptake (SUV_max_), mean standardized uptake (SUV_mean_), metabolic tumor volume (MTV) and total lesion glycolysis (TLG) of the primary tumor were calculated and compared between MSI-H and microsatellite stability (MSS). Predictive factors of MSI status were selected from metabolic parameters and clinicopathological profiles via a multivariate analysis.

**Results:**

Of 420 colorectal cancers, 44 exhibited a high incidence of MSI. Both MTV and TLG were significantly higher in MSI-H group compared with the MSS group (*P* = 0.004 and *P* = 0.010, respectively). Logistic regression analysis indicated that CRC with MSI-H were related to younger age (*P* = 0.013), primary lesion located at right hemi-colon (*P* < 0.001) and larger MTV on PET/CT imaging (*P* = 0.019). MTV more than 32.19 of colorectal cancer was linked to the presence of MSI (*P* = 0.019).

**Conclusion:**

Tumor metabolic burden were higher in MSI-H CRC which may be useful for predicting the MSI status of CRC patient and thus aid in determination of immunotherapy for patients with CRC.

## Introduction

Colorectal cancer (CRC) is one of the most common malignancies and major cause of cancer-related death worldwide [1, 2]. Despite rapidly evolving in diagnosis and treatment these years, the prognosis of metastatic CRC (mCRC) patients remains poor, with a median overall survival (OS) of approximately 30 months [3]. The progression of more novel and effective therapeutic strategies is an urgent unmet need for CRC patients. Several advances from the past few years indicate the potential to prolong patient survival through effectiveness of treatments based on an individual’s tumor-specific biomarkers [4–6]. Arguably, the most prominent examples are provided by the successes with anti-programmed cell death 1 (PD-1) in CRC contributed by the selection of microsatellite instability high (MSI-H) [7], which were granted approval by the U.S. Food and Drug Administration (FDA) for MSI-H mCRC patients in May 2017 [8]. Several prospective clinical trials in chemotherapy-resistant MSI mCRC have demonstrated a high disease control rate (DCR) and a favorable progression-free survival (PFS) with immune checkpoint inhibitors (ICPIs) [6, 9]. However, reported response rates (RR) of 23–69% and DCR of 47–89% to ICPIs in MSI-H CRC are likely reflecting patient and/or tumor heterogeneity [6, 10, 11]. Selecting CRC patients with MSI-H will be exceptionally useful in screening out patients most likely to benefit from immunotherapy to prolong patient survival and improve prognosis.

Currently, there are three major methods for detecting MSI status, including immunohistochemistry (IHC), polymerase chain reaction (PCR) and next-generation sequence (NGS) [12]. Although these analyses of CRC samples have been incorporated into routine clinical practice for the purpose of treatment algorithms, the heterogeneity of MSI-H status, poor DNA quality of biopsy samples, the invasive procedure, or/and the long experiment period may become limiting factors in their clinical application [13, 14]. Therefore, alternative noninvasive strategies could therefore be of good choice.

Fluorine 18 (^18^F) fluorodeoxyglucose (FDG) PET/CT is a valuable molecular imaging modality that is a less invasive tool in the management of patients with CRC [15] . Despite imaging techniques being critical in the preoperative workup for diagnosis or prognostication, some correlation exist between pretreatment image findings and genomic expression in malignant disease, such as c-Met status in gastric cancer [16] and PD-L1 expression in lung cancer [17]. Similarly, Chung et al. have shown MSI status to be correlated with ^18^F-FDG uptake in gastric cancer [18]. Thus, we hypothesized that the pretreatment ^18^F-FDG PET/CT might be a helpful tool for non-invasively inferring the MSI-H CRC patients.

Therefore, our purpose was to explore the relationship between metabolic parameters, clinicopathological profiles and MSI expression in CRC patients.

## Materials and methods

### Study design and patients

**Fig. 1:**
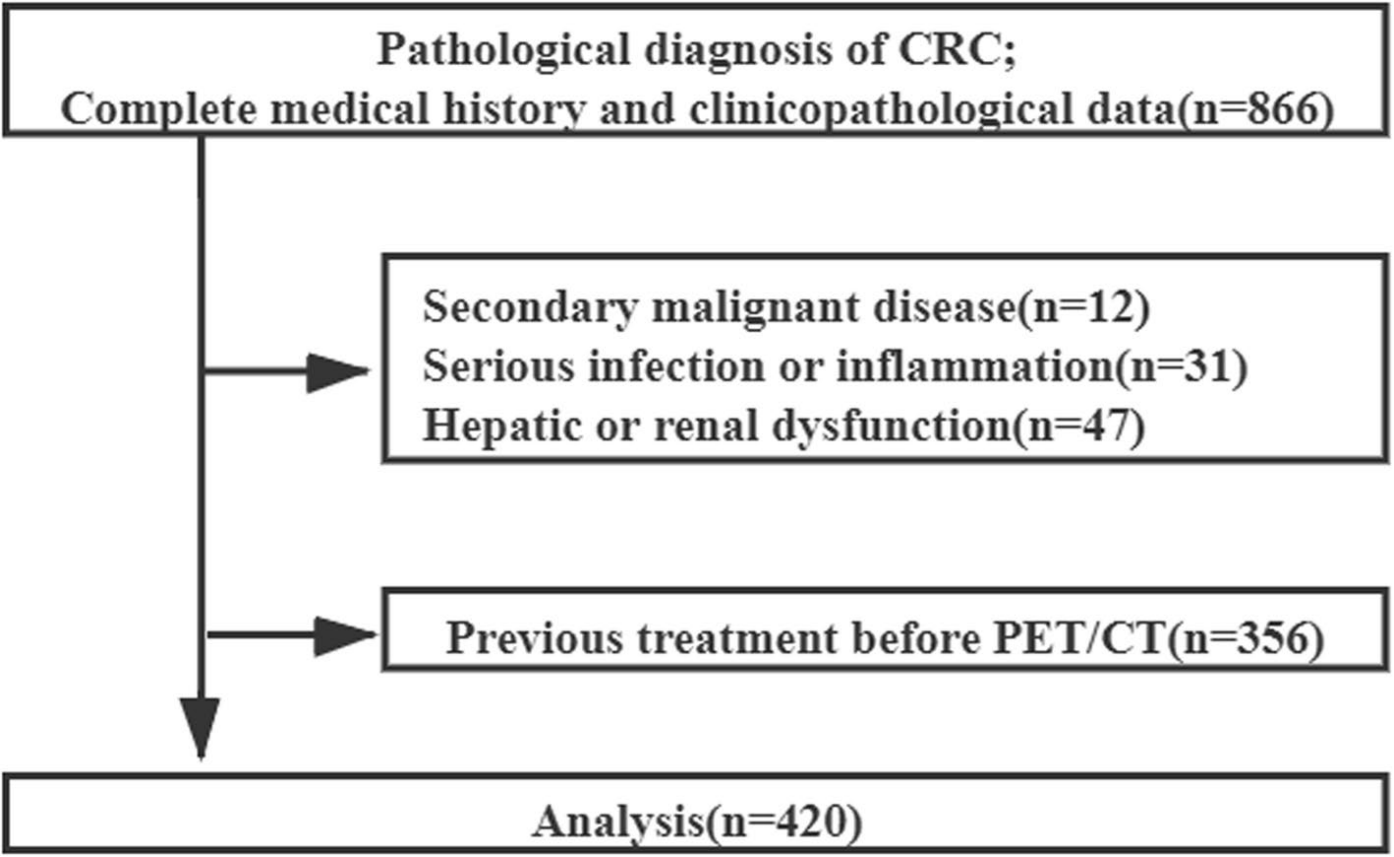
Flow diagram outlining criteria used for patient inclusion and exclusion

The Investigational Review Board of Peking University Cancer Hospital approved the present study (IRB number: 2022YJZ48). The requirement to acquire informed consent was waived owing to the Ethics Committee of Peking University Cancer Hospital. Retrospective data were collected with consecutive patients between January 2010 and March 2019 at Peking University Cancer Hospital based on the following criteria: (a) histologically proven CRC, (b) no prior treatment before ^18^F-FDG PET/CT scan, and (c) the presence of complete medical history and clinicopathological data. The exclusion criteria were (a) secondary malignant disease; (b) serious infection or inflammation; or (c) uncontrolled diabetes mellitus. Figure 1 shows the flowchart of selection criteria.

### ^18^F-FDG pet/CT

Whole-body acquisition was performed in 1–1.5 min/bed using a hybrid system (PHILIPS Gemini TF) that covered the area from the base of the skull to the upper thigh after intravenous injection of about 3.7 MBq/kg of ^18^F-FDG. All patients fasted for at least 6 h previously and presented with a blood glucose level lower than 10 mmol/L. Attenuation weighted ordered-subsets expectation maximization (AW-OSEM) iterative algorithm with 4 iterations and 8 subsets, Gaussian filter with 4.0 mm full width at half maximum (FWHM) and scatter correction were used for reconstruction [16].

### Quantitative PET parameter computation

Maximum standardized uptake volume (SUV_max_), mean SUV (SUV_mean_), metabolic tumor volume (MTV) and total lesion glycolysis (TLG) were calculated by selecting a Volume of Interest (VOI) in 3D mode using vendor-provided software at a PHILIPS EBW workstation. SUV(g/mL) = [measured activity concentration (Bq/mL)/ (injected activity [Bq]/body weight [kg] · 1000)], TLG(g) = SUV_mean_(g/mL) · MTV (cm^3^, [19]) (Units of these metabolic parameters are omitted for convenience below [20]). MTV was estimated for each primary CRC lesion with 40% of SUV_max_ as threshold and manual adjustment was applied when necessary to avoid nearby high physiologic uptake such as the bladder [21].

### Immunohistochemistry

Immunohistochemistry was carried out in an automated tissue staining system (ImmunoVision Technologies, Brisbane, CA). Briefly, formalin-fixed, paraffin-embedded blocks were cut into 4-μm-thick sections, deparaffinized in xylene and rehydrated, which also used for histopathologic determinations and tumor-infiltrating lymphocytes (TIL). Antigen retrieval was performed using EDTA (pH 8.0; Santa Cruz Biochemistry, Dallas, TX) in a pressure cooker for 3 minutes. The sections were incubated in 3% H_2_O_2_ solution for 10 minutes at room temperature to block endogenous peroxidase activity.

IHC was conducted using antibody to hMLH1 (ready-to-use, clone GM002; GeneTech, Shanghai, CHINA), hMSH2 (ready-to-use, clone RED 2; GeneTech, Shanghai, CHINA), hMSH6 (ready-to-use, cloneEP49; GeneTech, Shanghai, CHINA), and hPMS2 (ready-to-use, clone EP51; GeneTech, Shanghai, CHINA), CD8 (Leica Biosystems PA0183, mouse clone 4B11, ready-to-use formulation), and CD3 (Leica NCL-L-CD3–565, mouse clone LN10, diluted 1:100). Tumors were designated as a high incidence of MSI when at least one out of the four markers showed instability [13]. Microsatellite stability (MSS) was defined when no loss of expression was observed for any of these markers. ≥ 30 lymphocytes per 100 epithelial cells were considered as intra-epithelial lymphocytosis [22]. All CRC diagnoses were provided by specialty-trained intestinal pathologists.

### Statistical analysis

Statistical analysis was performed using SPSS software 23.0, (Inc.). Data are presented as median (range). Significant differences of variable characteristics between groups were compared by Mann-Whitney U-test for the continuous variables, and χ^2^ tests for the categorical variables. Receiver operating characteristic (ROC) curves were used to define optimal cut-off values for age, MTV and TLG using MedCalc software (Version 20.027). Logistic regression analysis was performed to determine the independent significant clinicopathological factors that showed a causal relationship with a dependent variable; a forward conditional method was used and the results are reported as hazard ratios (HR) and 95% confidence intervals (CI). Datasets were compared for patient demographic and clinical characteristics. *P* < 0.05 was considered statistically significant. Power analysis was performed using PASS software (Version 21.0.3).

## Results

### Patient characteristics

A total of 420 patients met our clinical and PET-based inclusion criteria and the details of clinicopathological characteristics are presented in Table 1. The mean age of CRC patients was 60 years (range 18–87 years), and 254 of these patients (60.5%) were men. The primary tumor was localized in the right hemi-colon in 118 (28.1%) patients, and moderately/highly differentiation was the most common histologic grade (approximately 85.9% of patients). The majority of patients were T_2–3_ (71.2%), N_1–2_ (58.6%) and M_0_ (79.3%) stage according to pathological results. On the basis of MSI status analysis of the primary tumor, 420 observational patients were classified into 2 groups: patient with MSI-H (44 patients, 10.5%) and MSS (376 patients, 89.5%). The median SUV_max_, SUV_mean_, MTV and TLG values for the primary lesion were 13.775 (4.10–54.73), 7.93 (2.81–22.70), 18.56 (range 1.34–391.04) and 141.25 (range 5.36–2634.39), respectively.

**Table 1:** Clinical characteristics of patients

Variable		Number of the subjects(***N*** = 420)
**Gender**	Male	254(60.5)
	Female	166(39.5)
**Age(y)**	< 52	100(23.8)
	≥52	320(76.2)
**Primary lesion**	Left hemi-colon	302(71.9)
	Right hemi-colon	118(28.1)
**Histologic grade**	poorly/moderately-poorly	56(14.1)
	moderately /highly	340(85.9)
**Mucinous carcinoma**	Yes	34(10.0)
	No	386(90.0)
**T stage**	T_2–3_	299(71.2)
	T_4_	121(28.8)
**N stage**	N_0_	174(41.4)
	N_1–2_	246(58.6)
**M stage**	M_0_	333(79.3)
	M_1_	87(20.7)
**MSI state**	Absent	376(89.5)
	Present	44(10.5)

The data presented are number (%) of patients

### Clinicopathological findings and metabolic parameters according to the MSI status

**Fig. 2:**
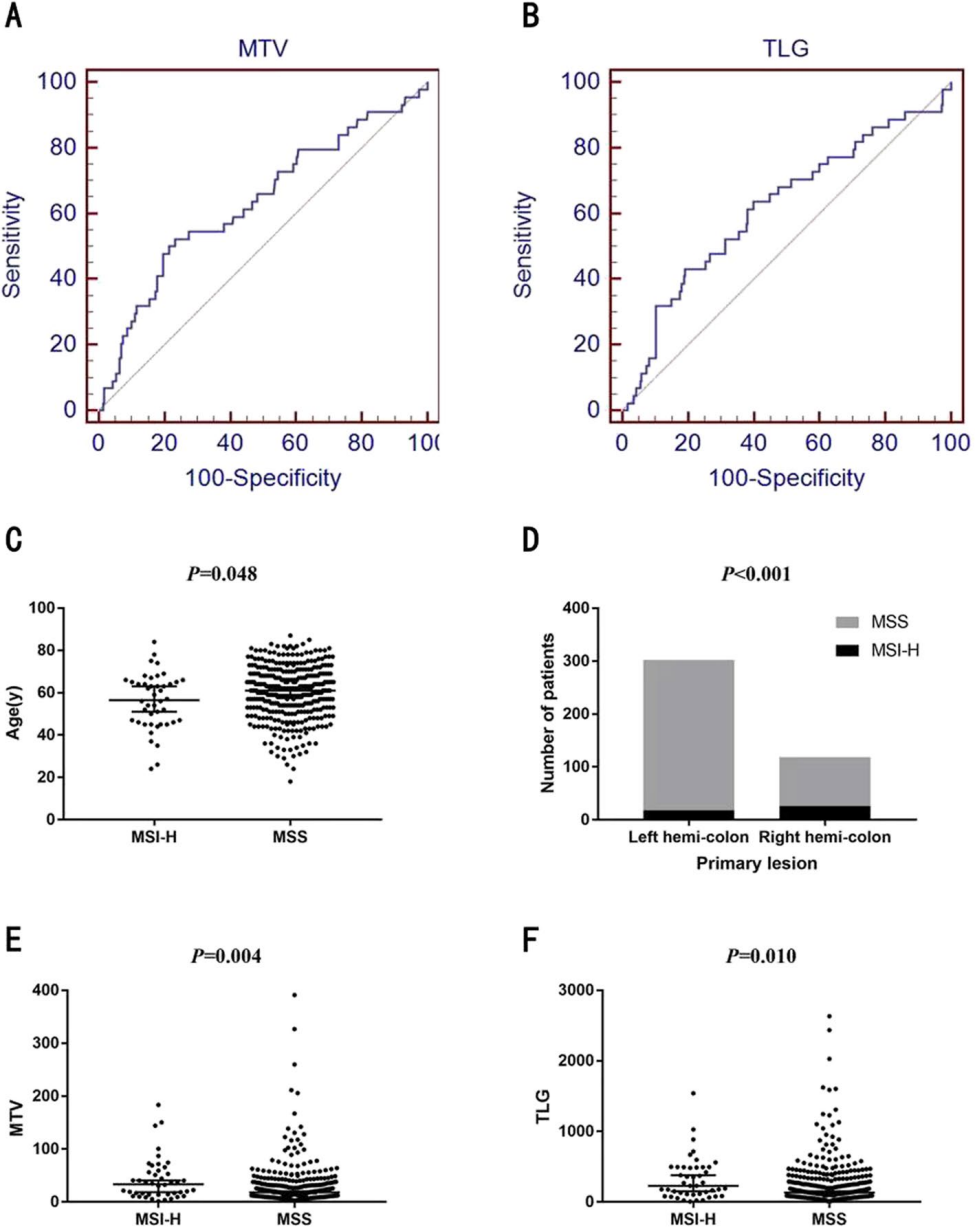
**A** ROC curve of MTV for predicting MSI-H. Sensitivity and specificity were 52.3 and 76.6% (area under curve (AUC) value = 0.633; *P* = 0.004). **B** ROC curve of TLG for predicting MSI-H. Sensitivity and specificity were 43.2 and 80.9% (area under curve (AUC) value = 0.619; *P* = 0.010). **C** Correlation between Age and MSI status in CRC. Younger patients prone to encounter in MSI-positive CRC [56.5(24–84) vs. 61(18–87); *P* = 0.048]. **D** Correlation between primary lesion and MSI status in CRC. MSS were located predominantly in the left hemi-colon (75.5%), whereas more than half of the MSI-H colorectal cancers were found in the right (59.1%, *P* < 0.001). **E** Correlation between MTV and MSI status in CRC. MTV was significantly higher in tumors with MSI-H than in those with MSS (33.60 vs.18.08; *P* = 0.004). **F** Correlation between primary tumor TLG and MSI status in CRC. TLG was significantly higher in tumors with MSI-H than in those with MSS (229.03 vs. 134.26; *P* = 0.010)

The correlations between patient characteristics and MSI status are summarized in Table 2. More than half of the MSI-H CRC were located in the right (59.1%), whereas MSS CRC were found predominantly in the left hemi-colon (75.5%) (*P* < 0.001) (Fig. 2). And younger patients prone to encounter in MSI-positive CRC [56.5(24–84) vs 61(18–87); *P* = 0.048; Table 2, Fig. 2]. MSI-H colorectal cancers exhibited significantly higher MTV (33.60) and TLG (229.03) values of the primary lesion than MSS ones (18.08, and 134.26, respectively; *P* = 0.004 and 0.010, respectively) (Fig. 2). However, no significant differences between the MSI-H and MSS groups were found in terms of gender, histologic grade, TNM category and SUVs (Table 2).

**Table 2:** Correlations between patient characteristics and MSI status in CRC patients

	CRC with MSI-H(***n*** = 44)	CRC with MSS(***n*** = 376)	***P*** value
Age, years, median, (range)	56.5(24–84)	61(18–87)	0.048*
Male: female ratio	29:15	225:151	0.436^#^
Primary lesion (right: left hemi-colon)	26:18	92:284	< 0.001^#^
Histologic grade (poorly/moderately-poorly: moderately /highly)	11:31	45:309	0.018^#^
Mucinous carcinoma (yes: no)	3:50	31:338	0.686^#^
T stage (T_2–3_: T_4_)	39:12	260:108	0.152^#^
N stage (N_0_: N_1–2_)	26:25	148:220	0.225^#^
M stage (M_0_: M_1_)	45:7	287:80	0.507^#^
SUV_max_, median(range)	13.81(5.97–47.06)	13.78(4.10–54.73)	0.502*
SUV_mean_, median(range)	7.29(3.67–16.22)	7.95(2.81–22.70)	0.319*
MTV, median(range)	33.60(1.34–183.42)	18.08(1.86–391.04)	0.004*
TLG, median(range)	229.03(5.36–1540.76)	134.26(7.25–2634.39)	0.010*

*SUV*_*max*_ the maximum standardized uptake values, *SUV*_*mean*_ mean standardized uptake values, *MTV* metabolic tumor volume, *TLG* total lesion glycolysis

*p* values were calculated using the *Mann–Whitney U-test and ^#^χ2 test

**Fig. 3:**
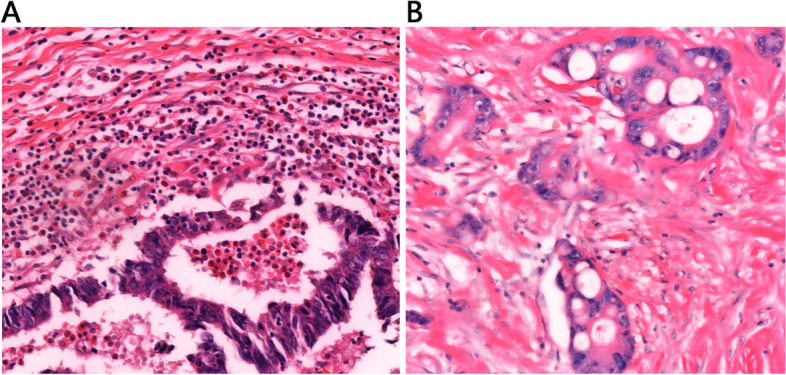
Representative pictures of intratumoral inflammatory cell infiltration by Hematoxylin-Eosin staining (× 20). **A** High density of inflammatory cell infiltration in MSI-H CRC. **B** Low density of inflammatory cell infiltration in MSS CRC

**Fig. 4:**
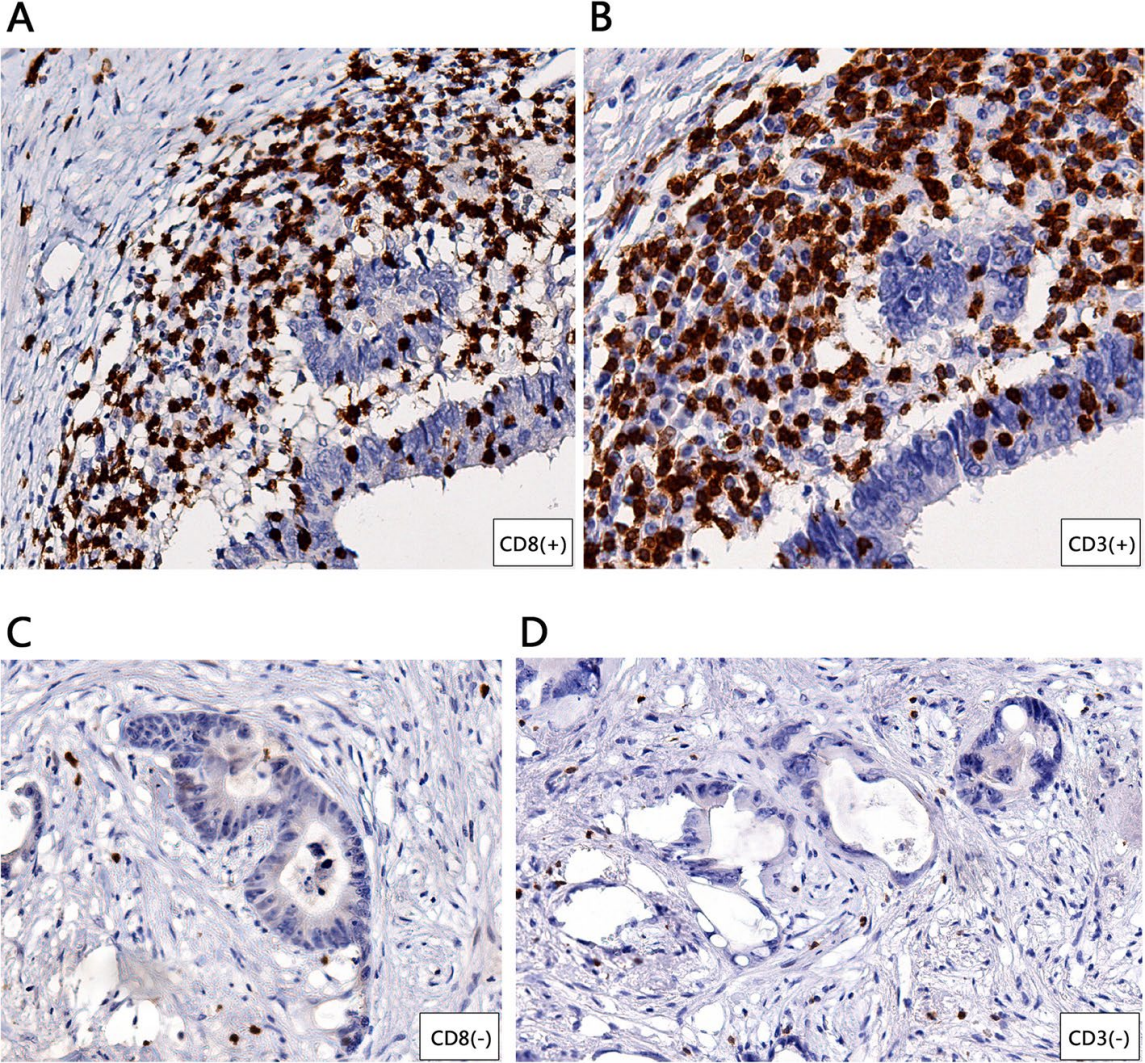
Representative Immunohistochemical detection of CD8 and CD3 (× 20) expression in slides of MSI-H CRC (**A**, **B**) and MSS CRC (**C**, **D**)

HE-staining results showed intratumoral inflammatory cell infiltration, and the TIL in the MSI-H group was significantly higher than in the MSS group (Fig. 3). IHC staining of CRC lesion showed that CD8+ were resided in the tumor stroma and the tumor epithelium (Fig. 4). These results can suggest that MSI-H group had higher metabolic burden, possibly by increased density of TILs.

### Predictive factors for the presence of microsatellite instability

**Fig. 5:**
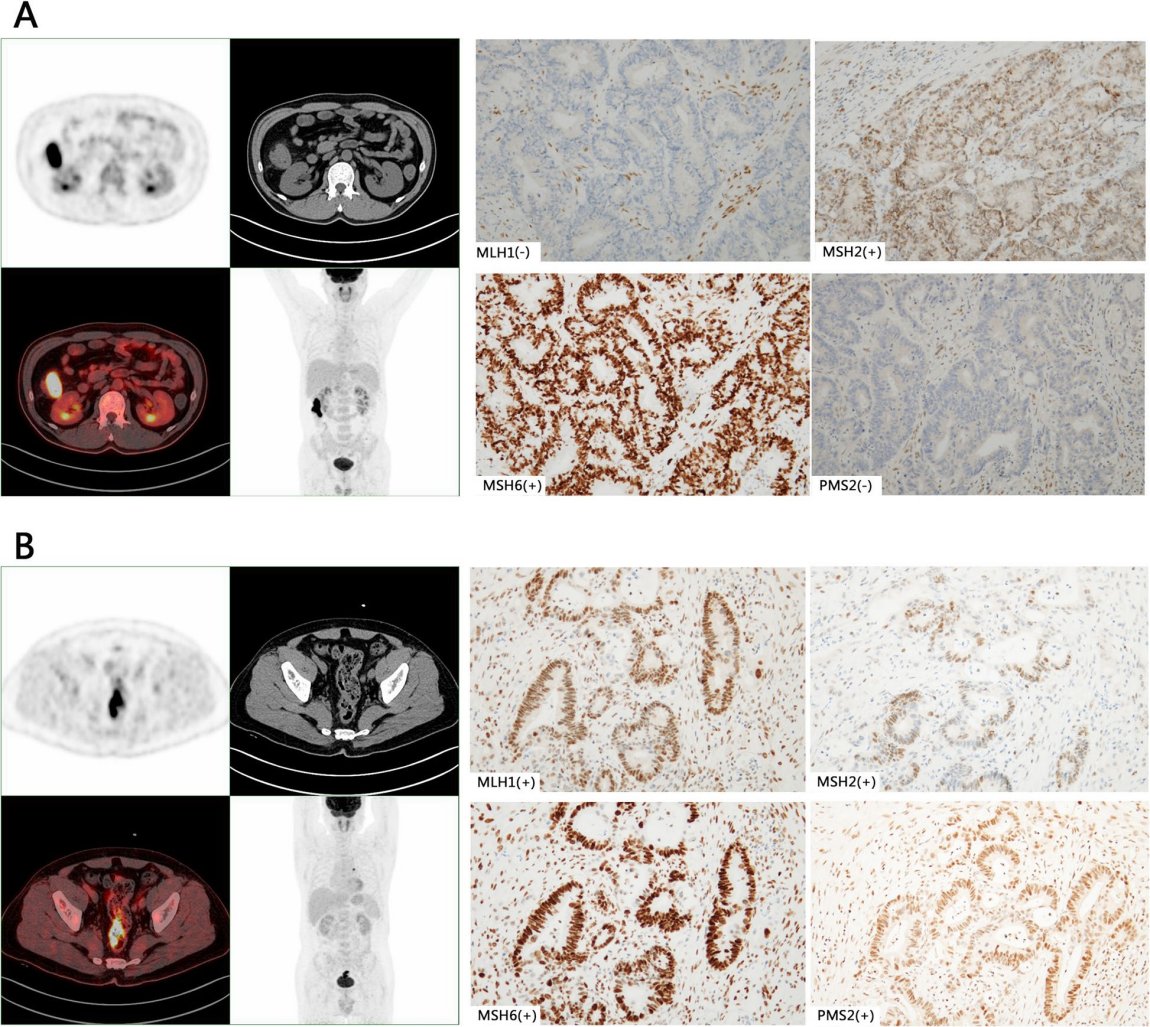
**A** a 45-year-old male had right colon cancer. ^18^F-FDG-PET/CT scans showed intense accumulation of ^18^F-FDG in the tumor (arrow; MTV,37.26; TLG,494.44). IHC analysis revealed MSI-H(MLH1(−) and PMS2(−)) after surgical resection. Scale bars, 100 mm. **B** a 55-year-old male had sigmoid colon cancer. ^18^F-FDG-PET/CT scans showed high accumulation of ^18^F-FDG in the tumor (arrow; MTV, 12.11; TLG,114.91). IHC analysis revealed MSS after surgical resection. Scale bars, 100 mm

We sought to determine the threshold of MTV and TLG for optimal differentiation between the MSI-H CRC group and the MSS CRC group. The optimal cut-off values of MTV and TLG were 32.19 (*P* = 0.004) and 352.72 (*P* = 0.010), respectively. Logistic analysis for microsatellite instability status revealed that right hemi-colon (HR 4.72, 95% CI 1.89–8.95; *P* < 0.001), age < 52y (HR 2.22, 95% CI 1.10–4.47; *P* = 0.026) and MTV ≥ 32.19 (HR 2.34, 95% CI 1.19–4.61; *P* = 0.014) were independent variables predicting MSI status (Table 3, Fig. 5).

**Table 3:** Logistic regression analysis for MSI in CRC patients

Variables	Odds ratio	95% confidence interval	***P*** value
Age (< 52 vs. ≥52 years)	2.22	1.10–4.47	0.026
Sex (male vs. female)	0.74	0.32–1.71	0.484
Primary lesion (right vs. left hemi-colon)	4.72	1.89–8.95	< 0.001
Histologic grade (poorly/moderately-poorly vs. moderately /highly)	0.65	0.24–1.75	0.391
Mucinous carcinoma (yes vs. no)	0.731	0.14–3.85	0.712
T stage (T_2–3_ vs. T_4_)	0.518	0.18–1.48	0.219
N stage (N_0_ vs. N_1–2_)	0.67	0.30–1.48	0.322
M stage (M_0_ vs. M_1_)	0.95	0.33–2.77	0.928
SUV_max_ (< 14.74 vs. ≥14.74)	0.96	0.37–2.48	0.935
SUV_mean_ (< 9.28 vs. ≥9.28)	0.44	0.15–1.31	0.140
MTV (≥32.19 vs. < 32.19)	2.34	1.19–4.61	0.014
TLG (≥352.72 vs. < 352.72)	1.44	0.49–4.26	0.512

## Discussion

The most important contribution of this study is the evaluation of MSI status of primary CRC lesions with a set of metabolic parameters (SUV, MTV, and TLG) on ^18^F-FDG PET/CT imaging to guided personalized medicine. The strong predictive value of higher MTV, younger age and right hemi-colon could be confirmed with MSI-H CRC. This study demonstrated the significance of MSI status in detecting CRCs using PET/CT imaging.

CRC can be categorized into two discrete groups on the basis of mutation patterns: tumors that have an MSI signature with high overall mutation burden (> 12 mutations per 10^6^ DNA bases) and tumors that have an MSS signature with a much lower mutation burden (< 8.24 mutations per 10^6^ DNA bases) [23]. MSI-H CRCs, approximately 10–15% of all sporadic CRCs [24, 25], are more common in younger patients (< 50 years), right-sided and poorly differentiated stage 2 CRCs [26–28]. MSI-H colorectal cancers (CRCs) have been shown to have a better overall prognosis compared with MSS cancers [29]. There is also evidence that MS-H is one of these important molecular markers which not only associated with resistance to 5-fluorouracil (5-FU) based chemotherapy particularly in stage II patients but also predicts the response of CRC to PD-1 blockade [10, 29–31]. Several PD-1 blockades, such as pembrolizumab and nivolumab, were granted by FDA for adult and pediatric patients with unresectable or metastatic microsatellite instability–high (MSI-H) tumors. Thus, identifying potentially susceptible subgroups is important and necessary for applying immunotherapy in CRC patients.

In the present study, we have added another piece in the big puzzle of immunotherapy. Indeed, by using ^18^F-FDG PET/CT, we demonstrated and interesting association between MSI status and elevated metabolic tumor burden. This is different from a recent study of Chung and colleagues [18] in gastric cancer, exhibiting that MSI-H tumors caused higher SUV_max_ on^18^F-FDG PET/CT imaging, that may because gastric cancer with MSI-H tended to be larger-sized and histologically heterogeneous, which was different from colorectal cancer. We found a positive relationship between MSI status and MTV in colorectal cancer, which was in agreement with the previous findings [32]. Liu et al. [32] retrospectively analyzed the pretreatment parameters of PET and reported the highest diagnostic performance of MTV in predicting MSI status in CRC despite its small sample size of 50 patients. However, the specific mechanism behind the correlation between metabolic tumor burden and MSI status is still unknown. Numerous publications have identified histologic features and heterogeneity which are more commonly seen in MSI-H CRCs. MSI-H tumor have high level of CD3+ and CD8+ TILs and a prominent inflammatory reaction (Crohn-like reaction) at the advancing edge of the tumor [28, 33]. Arguably, a high level of somatic mutations often occurred in MSI-H tumors, generating multiple neopeptides and eliciting a robust host immune response associated with increased density of TILs and upregulation of immune checkpoint expression (including PD-1, PD-L1, and CTLA-4, [34, 35]. Upregulation of PD-L1 was reported to be linked to the activation of mitogen-activated protein-kinase (MARK) and phosphoinositide 3-kinase (PI3K) signaling pathways as well as the hypoxia-inducible factor-1α (HIF-1α), an essential factor contributing to the elevated FDG uptake [36]. In present study, we found MSI-H CRC had large tumor size with a circumscribed/expansile growth pattern. Histology results revealed abundant lymphocytes infiltrate CRC lesions by conventional H&E stain. Staining with antibodies against CD3 and CD8 revealed the presence of T cells both within and at the invasive margin of the tumor. Increased density of TILs, a prominent inflammatory reaction, and activated HIF-1α may explain that significantly higher MTV (33.60) and TLG (229.03), reflecting the number of cells with abnormal metabolism, of primary MSI-H CRC than MSS ones (18.08, and 134.26, respectively; *P* = 0.004 and 0.010, respectively) in present study.

^18^F-FDG PET may play a pivotal role in guiding ICI treatment since there is a strong rationale suggesting that abnormally increased glucose metabolism is a hallmark of cancer associated with tumor aggressiveness and immune response [37–39]. In this study, we reviewed 8 clinicopathological indicators and 4 metabolic parameters of 420 CRC patients and tried to explore their correlation with MSI status. We found approximately 10% of all CRC and 8% of mCRC with MSI-H, predominantly located in the right hemi-colon (59.1%) among younger people (age < 52) in our cohorts. This is in line with reported studies [27, 40–42]. These results may be of significance for patients who would like to receive PD-1/PD-L1 blockades but are unable or unwilling to undergo biopsy for MSI status testing, and provide reference value to the immunotherapy.

Despite these strengths, our study has some notable limitations. First, this was a single-centre study; therefore, a future large-scale multicentred study should be conducted to validate our results. Second, our cohort included mainly locally advanced or mCRC, and it remains unclear whether the conclusion can apply in early stage CRC patients. Finally, even though our study found ^18^F-FDG PET/CT may have good prediction performance, it cannot replace pathologic testing for examining MSI status. Nevertheless, our study has demonstrated that the ^18^F-FDG PET/CT metabolic parameters in combination with clinicopathologic features is clinically relevant for guiding therapeutic strategies in patients with CRC.

## Conclusion

The MSI-H CRCs have higher MTV and younger age than the MSS types and mostly located in in the right hemi-colon in our large patient cohort. Our founding may serve as powerful indicators that identify patients with MSI status, those who cannot tolerate platinum-containing regiments but may benefit from immunotherapy such as anti-PD-1monoclonal antibody.

## Data Availability

The datasets used and/or analysed during the current study are available from the corresponding author on reasonable request.

## Abbreviations

CRC: Colorectal cancer
MSI-H: Microsatellite instability high
^18^F-FDG PET/CT: [^18^F]Fluorodeoxyglucose positron emission tomography/computed tomography
SUV_max_: Maximum standardized uptake value
SUV_mean_: Mean standardized uptake value
MTV: Metabolic tumor volume
TLG: Total lesion glycolysis
